# Thermal limits of wild and laboratory strains of two African malaria vector species, *Anopheles arabiensis* and *Anopheles funestus*

**DOI:** 10.1186/1475-2875-11-226

**Published:** 2012-07-06

**Authors:** Candice L Lyons, Maureen Coetzee, John S Terblanche, Steven L Chown

**Affiliations:** 1 Centre for Invasion Biology, Department of Botany and Zoology, Stellenbosch University, Private Bag X1, Matieland 7602, South Africa; 2 Malaria Entomology Research Unit, School of Pathology, Faculty of Health Sciences, University of the Witwatersrand, Johannesburg, South Africa; 3 Department of Conservation Ecology and Entomology, Stellenbosch University, Matieland, 7602, South Africa; 4 Current address: School of Biological Sciences, Monash University, Victoria, 3800, Australia

**Keywords:** Culicidae, Laboratory adaptation, Phenotypic plasticity, Thermal biology, Tolerance limits

## Abstract

**Background:**

Malaria affects large parts of the developing world and is responsible for almost 800,000 deaths annually. As climates change, concerns have arisen as to how this vector-borne disease will be impacted by changing rainfall patterns and warming temperatures. Despite the importance and controversy surrounding the impact of climate change on the potential spread of this disease, little information exists on the tolerances of several of the vector species themselves.

**Methods:**

Using a ramping protocol (to assess critical thermal limits - CT) and plunge protocol (to assess lethal temperature limits - LT) information on the thermal tolerance of two of Africa’s important malaria vectors, *Anopheles arabiensis* and *Anopheles funestus* was collected. The effects of age, thermal acclimation treatment, sex and strain (laboratory *versus* wild adults) were investigated for CT determinations for each species. The effects of age and sex for adults and life stage (larvae, pupae, adults) were investigated for LT determinations.

**Results:**

In both species, females are more tolerant to low and high temperatures than males; larvae and pupae have higher upper lethal limits than do adults. Thermal acclimation of adults has large effects in some instances but small effects in others. Younger adults tend to be more tolerant of low or high temperatures than older age groups. Long-standing laboratory colonies are sufficiently similar in thermal tolerance to field-collected animals to provide reasonable surrogates when making inferences about wild population responses. Differences between these two vectors in their thermal tolerances, especially in larvae and pupae, are plausibly a consequence of different habitat utilization.

**Conclusions:**

Limited plasticity is characteristic of the adults of these vector species relative to others examined to date, suggesting limited scope for within-generation change in thermal tolerance. These findings and the greater tolerance of females to thermal extremes may have significant implications for future malaria transmission, especially in areas of current seasonal transmission and in areas on the boundaries of current vector distribution.

## Background

Malaria affects large parts of Africa and Asia and is responsible for nearly 800,000 deaths annually. Despite interventions resulting in a reduction in global malaria mortality in the last 10 years [1], much concern still exists that in regions where malaria is either endemic, seasonal or has been present in the recent past, climate change might affect its presence and/or prevalence. Forecasts of the effects of climate change on the disease are controversial. Some sources indicate a possible spread of malaria at its current distribution margins [2], whilst others suggest that climate change will decrease the disease burden in many parts of its current range [3]. In southern Africa, malaria already presents a significant health risk [4], and how climate change will influence malaria incidence in this region [5-7], depends on several factors which remain poorly understood. These include the form of the change in climate [8], the environmental responses of the vectors [9,10], parasite-host interactions [11,12], and how interventions might interact with these changes [13,14].

In southern Africa, *Plasmodium falciparum*, the causative agent of cerebral malaria and the most common of the malarias in Africa, is transmitted by three primary vector species – *Anopheles gambiae*, *Anopheles arabiensis* and *Anopheles funestus*[15,16]. Current climate change forecasts for the parts of the region where malaria is endemic suggest an increase in both temperature and rainfall, both of which could increase the numbers of mosquitoes and hence the number of cases of the disease [17]. However, many factors remain to be clarified, including how the vectors will respond to such changed climatic conditions. Information on the response of vectors in southern Africa to a variety of conditions is necessary to forecast any change in malaria burden due to changing climates.

Understanding the likely future abundance and distribution of free-living organisms (including malaria vectors) usually involves some form of species distribution modelling, either using environmental niche modelling or a more mechanistic approach [10]. Both approaches have been used to estimate the impacts of climate change on mosquito vectors [18-20], and it has been suggested that a combination of the two can provide the most insight because both the fundamental and realized niches can be estimated (or a sound assessment made of all the factors influencing abundance and distribution) [10]. For mechanistic models, typically a range of basic physiological information is required, such as thermal tolerance limits, desiccation resistance and development rate [9,21].

Because many insect species show phenotypic plasticity [22], because the sexes often differ in their thermal response [23], and because tolerances may change with age, and age is an important feature of structured population models [24,25], these aspects should ideally be investigated too. A further complication is the fact that for many vectors, populations long-established in the laboratory are used for assessments, but laboratory adaptation might affect the outcome of the assays [26-28]. In consequence, these factors must be considered when providing information that can be used for mechanistic niche modelling.

For *Anopheles* mosquitoes, information on physiological tolerances required for such species distribution modelling is largely lacking. Of the three primary southern African malaria vectors, *An. gambiae* has been the most widely studied from this perspective, followed by *An. arabiensis*, but information on the physiological responses of *An. funestus* is largely absent (a review of the published information is available from the authors on request). Furthermore, the immature forms of *An. arabiensis* and *An. funestus* have rarely been considered [29].

Here, comprehensive assessments of the thermal tolerances of these species, their phenotypic responses to short-term changes in the thermal environment, and an estimate of the extent of laboratory adaptation of these thermal tolerance traits are provided. Additionally, information on the upper and lower lethal temperature limits of the larval and pupal stages of both species is provided. Finally, how climate change might affect vector populations, and hence malaria transmission is briefly considered.

## Methods

### Laboratory strains

Two long-established laboratory colonies held at the Vector Control Reference Unit in Johannesburg were used for all investigations of thermal tolerance. *Anopheles arabiensis* was taken from the KGB colony, originally established in 1975 from Kanyemba in the Zambezi Valley, Zimbabwe (R.H. Hunt, pers. comm.) and *An. funestus* from the FUMOZ colony established in 2000 from southern Mozambique [30]. These colonies are maintained under an insectary temperature of 25 °C (± 1 °C) and 80% relative humidity (verified using repeated measures with a Masons Hygrometer, Brannan, UK) with 12:12 light/dark cycle and 45 min dusk/dawn simulation. Larvae are fed a mixture of ground-up dog biscuits and yeast extracts and females are offered a blood meal three times weekly and allowed to lay eggs two to three times weekly. All adults are provided with a 10% sugar water solution *ad libitum*.

### Wild populations

*Anopheles arabiensis* females were collected from Malahapanga in the Kruger National Park, South Africa (22° 53.23 S, 31° 02.22 E) in October 2010. Wild *An. funestus* females were collected from villages surrounding the Maragra Sugar Estate in southern Mozambique (25° 27.41 S, 32° 46.59 E) in April 2011. Adult anophelines were collected using active-search techniques from inside huts or houses or from indoor animal dwellings using a flashlight and 30 cm glass aspirator. Females were transported back to the laboratory within three days for egg-laying in polystyrene cups with rough surfaces at a density of 20 females per 250 ml and were provided with a ball of cotton wool moistened with 10% sugar water solution. Egg batches from these females were kept separate until positive species identifications of the wild adults were made using standard PCR methods [31,32]. The progeny of at least 80 individual females was used to establish a laboratory colony of the wild strains, with the fifth to seventh generations being used in experiments on *An. arabiensis*, and the first generation used in experiments on *An. funestus*. Different generations were used as a result of the inherent difficulties associated with establishing *An. funestus* colonies compared with *An. arabiensis* colonies (R.H. Hunt, pers. comm.). These colonies were kept under the same conditions as the laboratory strains.

### Critical thermal limits (CTL)

Three age groups for each of the laboratory strains were used. *An. arabiensis* adults were 10-, 15- and 20-day olds, while *An. funestus* adults were 10-, 20- and 30-day olds. These ages were chosen because of the different lengths of the gonotrophic cycle and different adult longevities of the two species [30,33]. Only two adult age comparisons for the wild *An. arabiensis* strain (10- and 15-day olds) and wild *An. funestus* strain (10- and 20-day olds) were possible due to low colony numbers and the requirement to make assessments before 10 generations in the laboratory.

Between 20 and 40 individual males and females from all age groups were exposed to each of three acclimation treatments prior to CT determinations. Adult mosquitoes were acclimated for a period of five to seven days at 20 °C, 25 °C or 30 °C and a RH > 80% at either insectary conditions (25 °C) or using PTC-1 Peltier portable temperature control cabinets (Sable Systems, Las Vegas, Nevada, USA, 20 ± 1 °C and 30 ± 1 °C). Humidity in the insectary was checked using a Masons hygrometer (Brannan, UK). At 20 °C and 30 °C, humidity was maintained through the use of distilled water (checked using a Hygrochron i-button, DS 1923-F5, Maxim/Dallas Semiconductor, Sunnyvale, CA, USA). Each acclimation treatment was maintained on a 12L:12D cycle for the five or seven day period. Most insect species show acclimation responses in less than seven days [34]. Following these acclimation treatments, 10 individuals (comprising five individuals of each sex) per individual trial were placed into a double-jacketed insulated chamber connected to a programmable water bath (Grant LTC-12 Series, Grant Instruments, Ltd., Cambridge, UK). For each age group and acclimation treatment of each species, a total of four replicate trials were completed. CTmin experiments started at 20 °C while CTmax started at 25 °C, decreasing or increasing at a rate of 0.25 °C/min, respectively after an equilibration period of 10 min. While it has been shown that rate of temperature change can significantly alter the upper thermal tolerances of various insect species, the current rate was chosen as one comparable with many other studies [35]. The CTmin was regarded as the point where individuals displayed reduced motor function (ie, onset of spasms) and could not cling to the tip of a paint brush, while CTmax was regarded as the point where individuals displayed reduced motor function following a period of rapid flight [36]. At no point were individuals removed from the trial chambers for assessments of motor function (ie, individuals were continuously subjected to the thermal assay).

### Lethal temperature limits

Lethal temperature (LT) determinations of larvae and pupae, most appropriate for less mobile stages [37], for both species were carried out on six groups of 10 individuals each, per life stage (n = 60 per exposure temperature). The plunging technique was used instead of a ramping protocol [37,38]. Each replicate (ie, group of 10 individuals) was exposed for a period of two hours to temperatures ranging from −12 °C to 8 °C for LLT (lower lethal temperature) and from 34 °C to 44 °C for ULT (upper lethal temperature) in 2 °C increments to ensure that 0% and 100% survival of test individuals was recorded. A water temperature of 24 ± 0.5 °C was used as a control and survival at this temperature was 100%. Temperatures were maintained through the use of programmable water baths (Grant LTD-20 and GR150 R4 Series, Grant Instruments, Ltd., Cambridge, UK). Following the two-hour exposure, experimental groups were returned to water at 24 °C (± 1 °C) and survival was scored every 24 hours until either eclosion to adulthood or complete mortality occurred. Percentage survival was then scored as the percentage of the 10 individuals that eclosed. Larvae were fed daily on the same larval food as the colony strains.

Adult lethal temperature experiments were performed on five groups of 10 individual males and females each (n = 50 individuals per sex, per temperature), acclimated at only one temperature (25 °C, RH 80%). The three age groups (*An. arabiensis*: 10-, 15- and 20-days; *An. funestus* 10-, 20- and 30-days), were used in the upper lethal temperature and the LLT experiments, with the exception of the LLT determinations for *An. arabiensis* adults where only two age groups (10- and 15-day olds) were used due to unexpected mortality in the colony. Each replicate (ie, group of 10 individuals) was exposed to a given temperature in the range −6 °C to 16 °C for LLT determinations and 24 °C to 38 °C for ULT determinations, for a period of four hours to ensure that 0% and 100% survival temperature was measured for both LLT and ULT. This four-hour temperature exposure was chosen as an estimate of the length of time of the hottest period in the day, to which mosquitoes would be exposed, based on generalized daily temperature profile data which show that for many regions, including those of tropical Africa, high daytime temperatures are maintained for approximately four hours [39,40]. Experiments were conducted in a SANYO incubator (MIR-154, SANYO Electric Co. Ltd., Osaka, Japan). A temperature of 25 ± 1 °C was chosen as a control and survival at this temperature was close to 100%. Adults were immediately removed from the exposure temperature following the four-hour period, given sugar water and left to recover at 25 °C (± 1 °C) and relative humidity of 80%. Survival was scored as the percentage of the 10 adults still living, 24 hours after the experiment concluded.

### Data analysis

Normality and homogeneity of variances were examined using Shapiro-Wilk’s tests and Levene’s tests, respectively (Statistica v. 11, StatSoft, Tulsa, Oklahoma, USA). Some deviations from normality were observed, but the model assumptions were generally met (supplementary materials, Additional file 1) and the sample sizes sufficiently large to allow for the use of parametric general linear models (GLM) [41], as implemented in R (v. 2.13.1) (R Foundation for Statistical Computing, Vienna, Austria). The first model examined the effects of age, acclimation, sex and strain on the variables CTmin and CTmax for each species. Because significant effects of strain or an interaction with strain were found for CTmin/max, models were then run separately for each strain incorporating age, sex and acclimation as predictor variables. As an estimate of effect size, the mean percent deviation in CTmin/max from the grand mean per group was calculated by subtracting from each factor mean, the grand mean, and dividing this by the grand mean, multiplied by 100 to obtain a percentage (Additional file 2). The sign of this % deviation from the mean provides an indication of whether or not each factor had on average a lower (negative) or higher (positive) CTmin/max than the grand mean.

The mean (± S.E.) lethal temperature at which 50% of the sample population died (LT_50_) for each species and life stage, in relation to age and sex (for adults) was determined through the use of logistic regression with binomial distributions (logit link) in R (v.2.13.1). Using Hochberg’s GT-2 method as described in [42], lower and upper 95% confidence limits for each group were calculated using the means and standard errors obtained from logistic regression analyses. Mean LT_50_ (± 95% C.I.) for each group was plotted. Overlapping confidence intervals indicate no significant difference between groups.

## Results

### Critical thermal limits of *Anopheles funestus*

**Figure 1:**
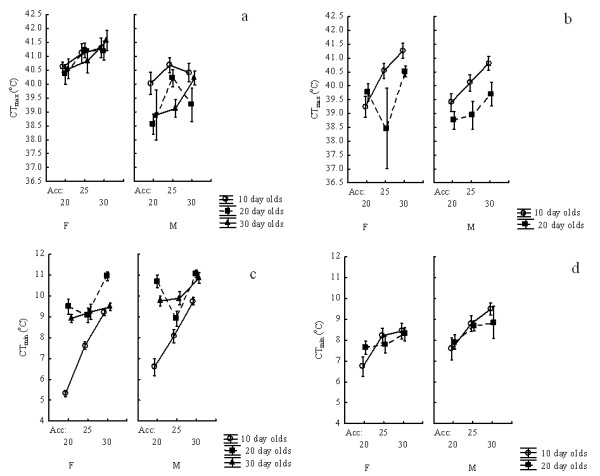
**The effects of age, sex and acclimation temperature on CTmax (a, b) and CTmin (c, d) in laboratory (a, c) and wild (b, d) strains of adult *Anopheles funestus.***

**Figure 2:**
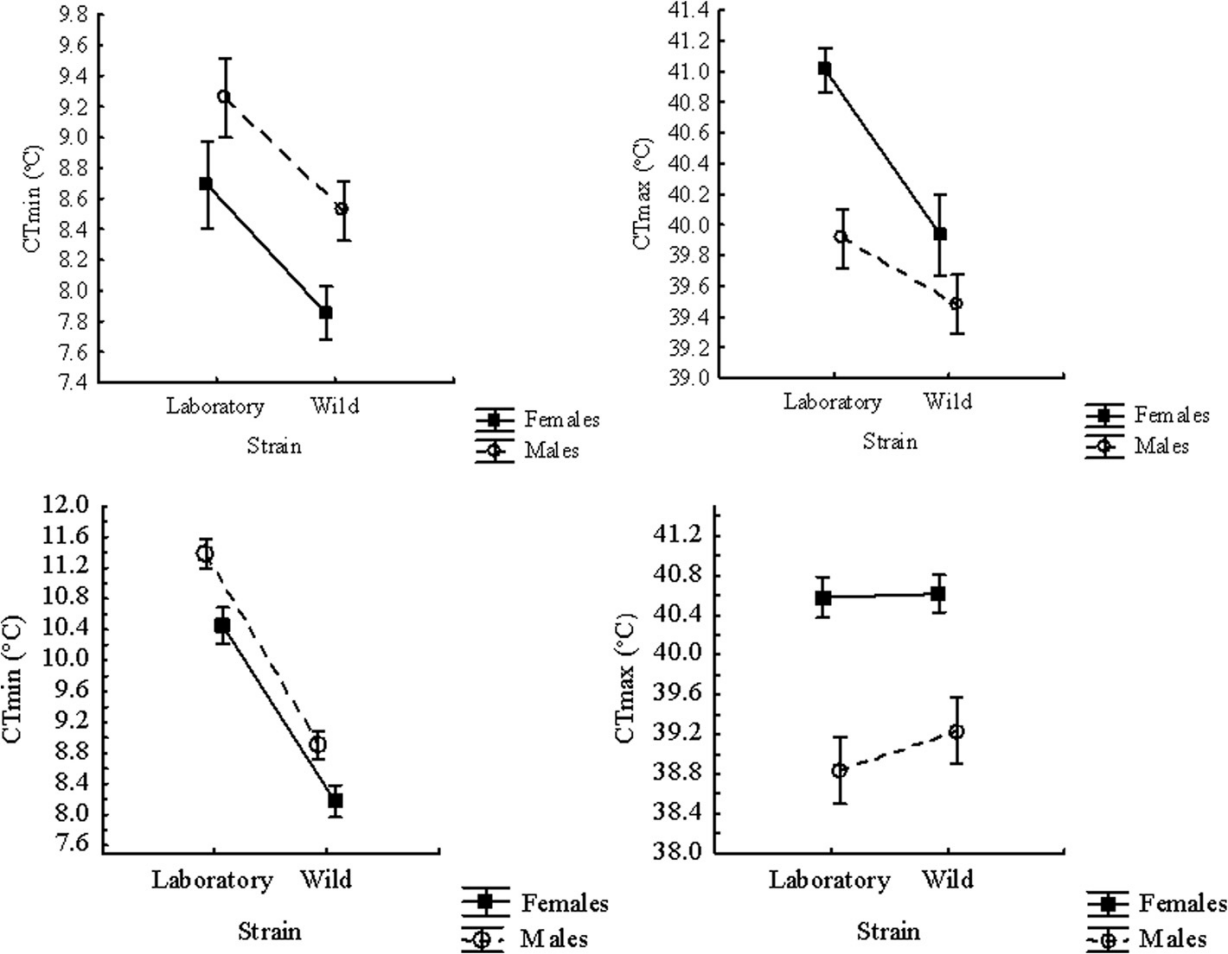
**Sex differences in CTmin (left) and CTmax (right) between the wild and laboratory strains of adult *Anopheles funestus *(top) and *Anopheles arabiensis *(bottom).**

No significant differences in CTmax were found between the wild and laboratory strains of *An. funestus* mosquitoes, and the interactions involving strain were generally not significant, except in a single case (Table 1, Figure 1). Only acclimation affected CTmax values significantly in both strains (Table 1), although the effect size was typically ≤2 °C, with the significant 3-way interaction between strain, age and acclimation not being clearly interpretable (Figure 1). However, it is clear that the overall acclimation response is less in the laboratory than wild strain in both males and females, explaining the significant two-way interaction between strain and acclimation. Clearly, some difference in the effects of acclimation, sex and age exists among strains and therefore the models were run separately for each strain (Table 2). Acclimation and age have much greater effects on CTmax in the wild than in the laboratory strains, with some differences in the interactions too. However, the total variation in CTmax was *c*. 3 °C (Figure 1, Additional file 2). Generally, higher acclimation treatments resulted in higher CTmax, and younger adults and females tend to have higher CTmax values (Figures 1 and 2). CTmin values differed between the *An. funestus* strains, which also showed significant differences in response to acclimation (Table 1). The strongest acclimation response was found in the colony strain and specifically in 10-day old males and females, whereas by comparison differences among other ages and among genders and acclimation treatments at other ages were much reduced (Figure 1). Maximum effect size of *c*. 4 °C was found following different acclimation treatments in 10-day old colony females (Figure 1, Additional file 2). When the models were run independently for the two strains it became clear that in each strain, sex, age and acclimation temperature had significant effects, but in somewhat different ways among strains, with the effects tending to be most pronounced in the laboratory strains (Table 3). Across the full set of treatments, the maximum difference in CTmin was *c*. 6 °C (Figure 1).

**Table 1:** **Outcomes of general linear models examining the effects of strain, sex, age, acclimation temperature and their interactions on CTmax (°C) and CTmin (°C) in adult *Anopheles funestus***

**Critical Thermal Limit**	**Effect**	**SS**	**df**	**F**	**P**
CTmax	Strain	3.14	1	3.13	0.078
	Sex	1.76	1	1.76	0.185
	Age	0.13	1	0.13	0.717
	**Acclimation**	**6.53**	**2**	**3.26**	**0.039**
	Strain*Sex	0.00	1	0.00	0.973
	Strain*Age	**23.87**	**1**	**23.81**	**< 0.0001**
	Sex*Age	1.68	1	1.68	0.196
	Strain*Acclimation	**10.07**	**2**	**5.02**	**0.007**
	Sex*Acclimation	1.52	2	0.76	0.469
	Age*Acclimation	0.72	2	0.36	0.699
	Strain*Sex*Age	**5.51**	**1**	**5.49**	**0.019**
	Strain*Sex*Acclimation	1.46	2	0.73	0.483
	Strain*Age*Acclimation	**23.43**	**2**	**11.68**	**< 0.0001**
	Sex*Age*Acclimation	1.07	2	0.53	0.588
	Strain*Sex*Age*Acclimation	2.74	2	1.37	0.256
CTmin	**Strain**	**6.59**	**1**	**12.91**	**< 0.0001**
	**Sex**	**16.13**	**1**	**31.59**	**< 0.0001**
	**Age**	**35.79**	**1**	**70.10**	**< 0.0001**
	**Acclimation**	**56.80**	**2**	**55.64**	**< 0.0001**
	Strain*Sex	0.84	1	1.65	0.200
	Strain*Age	**7.63**	**1**	**14.94**	**< 0.0001**
	Sex*Age	0.02	1	0.04	0.851
	Strain*Acclimation	**11.33**	**2**	**11.09**	**< 0.0001**
	Sex*Acclimation	**4.43**	**2**	**4.34**	**0.014**
	Age*Acclimation	**6.26**	**2**	**6.13**	**< 0.0100**
	Strain*Sex*Age	0.70	1	1.37	0.242
	Strain*Sex*Acclimation	2.48	2	2.43	0.089
	Strain*Age*Acclimation	0.66	2	0.65	0.523
	Sex*Age*Acclimation	0.84	2	0.82	0.441
	Strain*Sex*Age*Acclimation	2.98	2	2.92	0.055

**Table 2:** **Outcomes of general linear models examining the effects of sex, age, acclimation temperature and their interactions on CTmax (°C) of laboratory and wild strains of adult *Anopheles funestus***

**Strain**	**Effect**	**SS**	**df**	**F**	**P**
Laboratory	**Sex**	**3.45**	**1**	**4.16**	**0.042**
	**Acclimation**	**6.53**	**2**	**3.94**	**0.020**
	Age	0.74	2	0.45	0.640
	Sex*Acclimation	1.52	2	0.92	0.401
	Sex*Age	**8.69**	**2**	**5.24**	**0.006**
	Acclimation*Age	3.69	4	1.11	0.351
	Sex*Acclimation*Age	4.92	4	1.48	0.207
Wild	Sex	1.89	1	1.45	0.230
	**Age**	**42.85**	**1**	**32.84**	**< 0.0001**
	**Acclimation**	**41.7**	**2**	**15.98**	**< 0.0001**
	Sex*Age	4.09	1	3.14	0.078
	Sex*Acclimation	2.36	2	0.90	0.406
	Age*Acclimation	**38.18**	**2**	**14.63**	**< 0.0001**
	Sex*Age*Acclimation	**12.09**	**2**	**4.64**	**0.011**

**Table 3:** **Outcomes of general linear models examining the effects of sex, age, acclimation temperature and their interactions on CTmin (°C) of laboratory and wild strains of adult *Anopheles funestus***

**Strain**	**Effect**	**SS**	**df**	**F**	**P**
Laboratory	**Sex**	**16.13**	**1**	**42.05**	**< 0.0001**
	**Acclimation**	**202.23**	**2**	**263.63**	**< 0.0001**
	**Age**	**204.49**	**2**	**266.58**	**< 0.0001**
	Sex*Acclimation	**4.43**	**2**	**5.77**	**< 0.0100**
	Sex*Age	1.09	2	1.41	0.244
	Acclimation*age	**84.33**	**4**	**54.97**	**< 0.0001**
	Sex*Acclimation*Age	**8.43**	**4**	**5.49**	**< 0.0001**
Wild	**Sex**	**7.39**	**1**	**11.49**	**< 0.0001**
	**Acclimation**	**34.57**	**2**	**26.88**	**< 0.0001**
	**Age**	**8.62**	**1**	**13.39**	**< 0.0001**
	Sex*Acclimation	1.24	2	0.96	0.383
	Sex*Age	1.76	1	2.74	0.099
	Acclimation*Age	**10.18**	**2**	**7.92**	**< 0.0001**
	Sex*Acclimation*Age	2.54	2	1.97	0.142

### Critical thermal limits of *Anopheles arabiensis*

**Figure 3:**
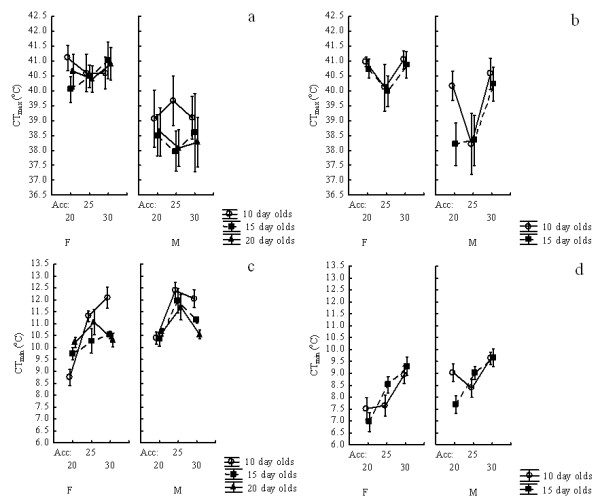
**The effects of age, sex and acclimation temperature on CTmax (a, b) and CTmin (c, d) in laboratory (a, c) and wild (b, d) strains of adult *Anopheles arabiensis.***

In *An. arabiensis*, with the exception of two-way interactions between sex and age, and sex and acclimation treatment, as well as several three-way interactions between strain, sex, age and acclimation treatment, no other effects on CTmax were significant, and especially not the main effects in the model (Table 4). It does appear that females have higher CTmax values than males (Figure 2), but these effects were not readily distinguished in the full model. When the models were implemented separately for each strain, the sex effect was significant (Table 5, Figure 2), as was the effect of acclimation for the wild strain, largely reflecting the large effect of the 30 °C acclimation treatment on 15-day old males (Figure 3). By contrast, age and various interactions did not have significant effects on the laboratory strain. The overall range of CTmax values was *c*. 3 °C (Figure 3).

**Table 4:** **Outcomes of general linear models examining the effects of strain, sex, age, acclimation temperature and their interactions on CTmax (°C) and CTmin (°C) for wild *versus *laboratory strains of adult *Anopheles arabiensis***

**Critical Thermal Limit**	**Effect**	**SS**	**df**	**F**	**P**
CTmax	Strain	0.14	1	0.06	0.799
	Sex	6.84	1	3.11	0.079
	Acclimation	11.23	2	2.55	0.079
	Age	0.68	1	0.31	0.580
	Strain*Sex	7.33	1	3.33	0.069
	Strain*Acclimation	5.08	2	1.15	0.316
	Sex*Acclimation	**13.89**	**2**	**3.15**	**0.044**
	Strain*Age	3.38	1	1.54	0.216
	Sex*Age	**15.39**	**1**	**6.99**	**0.009**
	Acclimation*Age	0.04	2	0.01	0.990
	Strain*Sex*Acclimation	**19.85**	**2**	**4.51**	**0.012**
	Strain*Sex*Age	**13.9**	**1**	**6.31**	**0.012**
	Strain*Acclimation*Age	5.4	2	1.23	0.294
	Sex*Acclimation*Age	12.49	2	2.84	0.060
	Strain*Sex*Acclimation*Age	**26.43**	**2**	**5.99**	**0.003**
CTmin	**Strain**	**15.01**	**1**	**24.97**	**< 0.0001**
	**Sex**	**22.95**	**1**	**38.19**	**< 0.0001**
	**Acclimation**	**24.57**	**2**	**20.45**	**< 0.0001**
	**Age**	**3.14**	**1**	**5.22**	**0.023**
	Strain*Sex	0.08	1	0.13	0.719
	Strain*Acclimation	**32.84**	**2**	**27.32**	**< 0.0001**
	Sex*Acclimation	**4.35**	**2**	**3.62**	**0.028**
	Strain*age	**12.17**	**1**	**20.25**	**< 0.0001**
	Sex*Age	**3.04**	**1**	**5.06**	**0.025**
	Acclimation*Age	**11.17**	**2**	**9.29**	**< 0.001**
	Strain*Sex*Acclimation	3.35	2	2.78	0.063
	Strain*Sex*Age	0.19	1	0.31	0.575
	Strain*Acclimation*age	**40.69**	**2**	**33.85**	**< 0.0001**
	Sex*Acclimation*Age	0.86	2	0.71	0.490
	Strain*Sex*Acclimation*Age	2.41	2	2.01	0.135

**Table 5:** **Outcomes of general linear models examining the effects of sex, age, acclimation temperature and their interactions on CTmax (°C) of laboratory and wild strains of adult *Anopheles arabiensis***

**Strain**	**Effect**	**SS**	**df**	**F**	**P**
Laboratory	**Sex**	**42.42**	**1**	**15.93**	**< 0.0001**
	Acclimation	3.61	2	0.68	0.508
	Age	15.50	2	2.91	0.056
	Sex*Acclimation	6.18	2	1.16	0.314
	Sex*Age	2.33	2	0.44	0.645
	Acclimation*Age	14.79	4	1.39	0.237
	Sex*Acclimation*Age	13.67	4	1.28	0.276
Wild	**Sex**	**6.59**	**1**	**3.90**	**0.049**
	**Acclimation**	**10.89**	**2**	**3.23**	**0.042**
	Age	0.52	1	0.31	0.579
	Sex*Acclimation	**11.08**	**2**	**3.28**	**0.039**
	Sex*Age	**15.54**	**1**	**9.20**	**0.003**
	Acclimation*Age	0.06	2	0.02	0.983
	Sex*Acclimation*Age	**11.01**	**2**	**3.26**	**0.040**

CTmin responded strongly to acclimation treatments, and age, sex and strain were also all significant in *An. arabiensis* (Table 4). The wild strain tended to have lower CTmin values than the laboratory strain, while 10-day old females in the laboratory colony showed the strongest response to acclimation (Table 6, Figure 3), just as was the case in *An. funestus*. In the wild strain, females tended to have a lower CTmin than males (Figure 2), and acclimation had a strong, generally linear effect on CTmin (Table 6, Figure 3). However, in the laboratory strain, although all of the main effects and interactions were significant (Table 6), the responses were non-linear among acclimation treatments, and the variation among age groups at a given acclimation ≤ 1.5 °C (Figure 3). Overall, among strains, ages, sexes and acclimation treatments the variation in CTmin was *c*. 5 °C (Figure 3, Additional file 2).

**Table 6:** **Outcomes of general linear models examining the effects of sex, age, acclimation temperature and their interactions on CTmin (°C) of laboratory and wild strains of adult *Anopheles arabiensis***

**Strain**	**Effect**	**SS**	**df**	**F**	**P**
Laboratory	**Sex**	**26.89**	**1**	**49.16**	**< 0.0001**
	**Acclimation**	**122.67**	**2**	**112.1**	**< 0.0001**
	**Age**	**22.55**	**2**	**20.60**	**< 0.0001**
	Sex*Acclimation	**14.65**	**2**	**13.39**	**< 0.0001**
	Sex*Age	**8.67**	**2**	**7.92**	**< 0.0001**
	Acclimation*Age	**63.03**	**4**	**28.79**	**< 0.0001**
	Sex*Acclimation*Age	**11.42**	**4**	**5.22**	**< 0.0001**
Wild	**Sex**	**22.95**	**1**	**34.29**	**< 0.0001**
	**Acclimation**	**24.57**	**2**	**18.36**	**< 0.0001**
	**Age**	**3.14**	**1**	**4.69**	**0.031**
	Sex*Acclimation	**4.35**	**2**	**3.25**	**0.041**
	Sex*Age	**3.04**	**1**	**4.55**	**0.034**
	Acclimation*Age	**11.16**	**2**	**8.34**	**< 0.001**
	Sex*Acclimation*Age	0.86	2	0.64	0.527

### Lethal temperature limits

**Figure 4:**
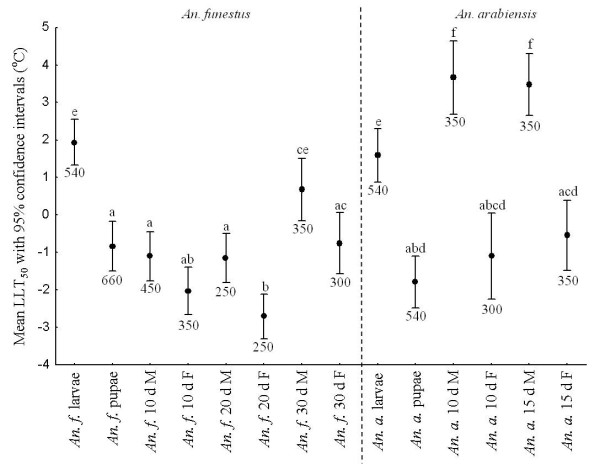
**Lower lethal temperatures for 50% of the sample population (LLT**_**50**_**) ± 95% confidence intervals for *Anopheles funestus *(left of the dashed line) and *Anopheles arabiensis *(right of the dashed line) larval, pupal and adult stages.** LLT_50_ data for adults include the influence of sex and age for both species. Differences in lower case letters indicate significant differences between groups, within and amongst species, while numbers below each line indicate sample size. Adults were exposed to temperature treatments for a period of four hours and larvae and pupae, for a period of two hours.

Lower lethal temperature (LLT) in *An. funestus* was approximately −1 °C to −2 °C for all stages and age groups examined, with the exception of the larvae (mean ± 95% C.I., 1.94 °C ± 0.62 °C), and 30-day old adult males (mean ± 95% C.I., 0.68 °C ± 0.83 °C), which were less tolerant of low temperature (Figure 4). In *An. arabiensis*, the situation was similar, with larvae likewise showing the least tolerance of low temperatures (mean ± 95% C.I., 1.59 °C ± 0.71 °C), and adult males being the least resistant of all groups (10-day old males mean ± 95% C.I., 3.66 °C ± 0.98 °C; 15-day old males mean ± 95% C.I., 3.48 °C ± 0.83 °C). Lower lethal limits in the adults were generally 8-11 °C less than the CTmin. The full range of LLT values spanned *c.* 6 °C (Figure 4).

**Figure 5:**
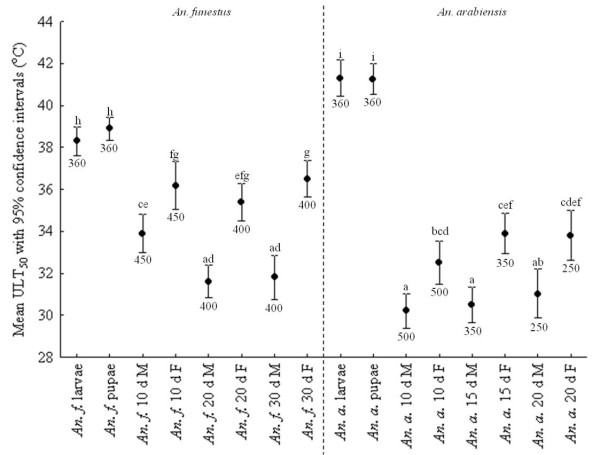
**Upper lethal temperature for 50% of the sample population (ULT**_**50**_**) ± 95% confidence intervals for *Anopheles funestus *(left of the dashed line) and *Anopheles arabiensis *(right of the dashed line) larval, pupal and adult stages.** ULT_50_ data for adults include the influence of sex and age for both species. Differences in lower case letters indicate significant differences between groups, within and amongst species, while numbers below each line indicate sample size. Adults were exposed to temperature treatments for a period of four hours and larvae and pupae, for a period of two hours.

Upper lethal temperatures (ULT) across the full range of stages, ages and species varied by *c*. 11 °C. In both species, larvae and pupae had the highest ULT, with *An. arabiensis* having more tolerant immature stages than *An. funestus* (Figure 5). Females of both species tended to have higher ULT than males, with the most heat sensitive group being the males of *An. arabiensis*. The lethal temperature estimates were typically 8-10 °C lower than the CTmax estimates, indicating a much reduced scope for long-term tolerance of high temperature in the adults.

## Discussion

Laboratory colonies are used for a wide range of investigations of insect responses to changing environmental conditions. These include investigations of the responses of mosquitoes to various thermal conditions (eg, [43]), and to pathogens and insecticides [44]. However, as has now been demonstrated in a range of arthropod taxa, laboratory adaptation and acclimation can take place rapidly, affecting some traits, but not others and affecting sexes differentially [26-28]. In consequence, extrapolations to the field situation, such as is required for mechanistic niche modelling or assessments of the outcomes of control interventions, may be compromised, making estimations of the extent of differences among laboratory and field strains essential.

The current results indicate that differences in mean CTmin or CTmax among the wild and laboratory strains of *An. arabiensis* and *An. funestus* typically did not exceed 2 °C. In most instances differences between strains were approximately 1 °C. The 2 °C difference among wild and laboratory strains was observed for CTmin in the longest-lived colony (35 years) of *An. arabiensis* and might indicate a loss of thermal tolerance after extensive exposure to constant laboratory colony conditions. Differences in the acclimation responses between the wild and laboratory strains were also evident. However, the range of acclimation responses over all treatments was similar for both strains except in younger females of the laboratory colonies. Thus, results suggest that although caution is required when extrapolating laboratory thermal tolerance data to the field, as is recommended for other aspects of malaria biology (eg, [45]), at least for the species examined here, using thermal tolerance data from laboratory colonies will provide a reasonable approximation of expected responses in the field (but see also [28]).

Other biologically significant sources of variation in thermal tolerance limits, especially in the context of understanding and forecasting responses to environmental change, are those associated with age, sex and short-term responses to change (phenotypic plasticity) [22,25,46]. Several recent studies have shown that upper lethal limits or limits to activity in insects and other ectotherms are typically much less variable, both among populations and species, and over time (through plasticity or responses to selection) than are lower limits [47-49]. The same pattern was found here for the adults and in addition the extent of variation amongst the age groups in CTmax and ULT tended to be fairly narrow. Thus, whilst increasing temperatures may benefit the species in cooler areas (contributing perhaps to rising malaria incidence as is the case in East Africa, eg, [50]), where they are close to their thermal limits rising temperatures may act to suppress populations. Indeed, constrained upper thermal limits may be the mechanistic basis, together with the thermal sensitivity of immature development (see [43], unpublished data), for the forecast range declines of *An. gambiae* and *An. arabiensis* in northern and west Africa and increases in south-eastern Africa [51]. In this respect, males might be more sensitive than females given the 1-2 °C difference in CTmax, which may well be associated with the blood-feeding habits of females [52].

Transmission of malaria is dependent on the effects of ambient temperature on the *Plasmodium* parasite, and on the effect of ambient temperature on the vector species. Lower limits to *Plasmodium* development are *c*. 16 °C. However, although parasite development rate increases with increasing temperatures, temperatures above *c.* 30 °C are detrimental to parasite development and could therefore, have consequences for transmission [11]. Transmission of malaria is also dependent on the ability of adults to withstand high temperatures [53] and the greater sensitivity of older mosquitoes to high temperatures, as found here in most cases, may cause female death before the parasite migrates to the salivary glands and can be transmitted. The complexity of this interaction between the sensitivity to temperature of malaria parasites and their vectors has been noted previously [11]. In consequence, rising temperatures may not only reduce mosquito population densities, but also the extent to which the malaria parasite is transmitted. The dynamics of this interaction are likely to be complicated by the habits of the vector (indoor or outdoor species), temperature variability, and the nature of the host-parasite interaction [12,50]. Nonetheless, the finding that temperature sensitivity, at least for critical limits, increases with age is in keeping with other studies of thermal responses in insects [25]. For mosquitoes, mortality is highly age- and infection-dependent [54]. Even in the absence of *Plasmodium* ookinetes, mortality of females increases with an increase in age, suggesting the potential for female anophelines to senesce [54]. As a confounding factor to malaria transmission, the presence of large numbers of parasite ookinetes in the mosquito midgut greatly increases the mortality experienced within a population and reduces overall mosquito longevity [54] adding to the potential for reduced overall mosquito populations, and hence, the potential for reduced malaria transmission, with increasing environmental temperatures as a consequence of climate change.

Variation found among the lethal and critical thermal limits for the two anopheline species is typical of that found in a range of other taxa [46,55]. Activity tends to cease well before the lower lethal limit in adults, whilst the upper lethal limits tend to be somewhat lower than the short-term tolerances represented by CTmax. The latter may in part be explained by the differences between the two techniques used to measure these variables and the rate at which temperature was changed during the ramping method used for CTmax estimation. Slower rates often, though not always, result in lower CTmax values [35,56]. Nonetheless, these thermal traits might also be under different genetic control [57]. Irrespective, it is clear that the most pronounced differences in ULT were found among the stages, with the immatures having ULTs 2-10 °C higher than those of the adults. Such among-stage variation is common in other insects and usually reflects their exposure to different conditions [25]. For *An. arabiensis* and *An. funestus*, as with many other species where the adults are more mobile than the immatures, greater tolerance to high temperatures can be expected in the immature stages. Behavioural regulation is more straightforward for a highly mobile individual living in air than for a much less active individual living in a thermally conductive medium such as water (see also [58]). In particular, the adults of both species are highly anthropophilic, with *An. arabiensis* displaying more exophilic behaviour than *An. funestus*[15,59]. This behaviour of the adults, combined with their mobility, means that they are able to escape unfavourable temperatures and make use of indoor-resting behaviour during the hottest or coldest parts of the day [60]. However, behavioural avoidance of temperature extremes is likely limited for larvae of *An. funestus* and is probably largely absent for larval *An. arabiensis*, owing to their breeding habits (see Table 7).

**Table 7:** **Differences in biology between *Anopheles arabiensis *and *An. funestus *[**[15]**]**

	***Anopheles arabiensis***	***Anopheles funestus***
**Southern African distribution**	Present in South Africa in the low-lying north-eastern areas	Absent from South Africa at present, but occurs in southern Mozambique
**Habitat type**	Arid-adapted, areas as low as 40% relative humidity, environmental temperatures as high as 50 °C	“Tropical species”, requires more humid environment, environmental temperatures up to 40 °C
**Breeding sites**	Shallow, temporary pools <0.5 m deep eg, hoof prints, tyre tracks	Swamps, slow-flowing streams, deep and vegetated water bodies
**Behaviour**	Exophilic and endophilic, feeds on cattle and humans	Endophilic, prefers to feed on humans

In absolute terms, the larvae of both *An. arabiensis* (ULT_50_*c*. 41 °C) and *An. funestus* (ULT_50_*c*.38 °C) were able to survive higher temperatures (to eclosion) than are those of *An. gambiae s.s.* (ULT_50_*c*.32 °C) [61], although generally the lethal limits were within the range found for anophelines [62-65]. *Anopheles arabiensis* breeds in shallow, temporary pools or puddles, while *An. funestus* prefers to breed in semi-permanent to permanent water bodies [15] (Table 7). The smaller water bodies are likely to show much greater thermal variation than the latter simply on the grounds of volume alone, and are also likely to offer less opportunity for microhabitat selection. Thus, the high upper thermal tolerances of *An. arabiensis* in the immature stages are not unexpected. Nonetheless, how the lethal limits determined here relate to thermal limits to development over the entire immature stage, given that the latter are typically narrower than the former [57] needs to be explored, especially in determining the environmental limits to distribution both at the upper and lower temperature extremes. Interactions between changing climates and lower development limits may account for forecasts of expansion of *An. arabiensis* into cooler areas as climates warm [51], given that such interactions can reasonably account for current coarse-scale distributions of *An. gambiae s.s.*[43]. Furthermore, interactions between climate and upper development limits may change the seasonality of occurrence or lead to range limitation, depending on interactions with rainfall (see eg [66]). Investigations of the relationship between lethal and development limits for both *An. arabiensis* and *An. funestus* are currently underway (unpublished data), and should provide insights into changing distribution patterns and the extent to which they match those forecast on the basis of environmental niche modelling alone (see [9]).

## Conclusions

This study has shown that with the necessary caution, laboratory colonies provide an initial basis for investigating physiological tolerances of *An. arabiensis* and *An. funestus* to both high and low temperatures. In addition, it suggests that limited variation in upper thermal limits may well account for forecasts of declining distributions in already warm areas as temperatures rise, whilst sensitivity of development may be more significant a limiting factor in cool areas, given low, lower lethal limits. Finally, this study has demonstrated substantial physiological differences in tolerance between two of the main malaria vectors in southern Africa, which will have to be taken into account when forecasting responses to environmental change of all kinds, including the ways in which water bodies are manipulated to account for expected changes in rainfall regimes.

## Competing interests

The authors declare that they have no competing interests.

## Authors’ contributions

CLL, MC, JST and SLC designed the research. CLL collected the data. CLL and SLC analysed the data. CLL, MC, JST and SLC wrote the manuscript. All authors read and approved the final manuscript.

## Supplementary Material

Additional file 1 **Results from a Shapiro-Wilk's test for normality and Levene’s test for homogeneity of variance for all groups and each group separately for *Anopheles arabiensis *and *Anopheles funestus*.** Click here for file

Additional file 2 **Percentage deviation from the mean critical thermal minimum (CTmin) and maximum (CTmax) per group, per strain for *Anopheles funestus *and *Anopheles arabiensis*.** Click here for file
