# Supplementary material for: Thermal limits of wild and laboratory strains of two African malaria vector species, Anopheles arabiensis and Anopheles funestus
Source: Malar J. 2012 Jul 6;11:226. doi: 10.1186/1475-2875-11-226 (PMC3507762; doi:10.1186/1475-2875-11-226)
Supplement: Additional file 1 — Results from a Shapiro-Wilk's test for normality and Levene’s test for homogeneity of variance for all groups and each group separately for Anopheles arabiensis and Anopheles funestus. [file 1475-2875-11-226-S1.pdf]

Additional file 1. Results from a Shapiro-Wilks test for normality and Levene's test for homogeneity of variance for all groups and each group separately for *Anopheles arabiensis* and *Anopheles funestus*.

Species and trait	Group	Shapiro-Wilks	Group	df	Levene's
An. arabiensis CTmin (˚C)	ALL	W = 0.99, P = 0.009	ALL	678	F = 3.64, P < 0.0001
	Laboratory strain	W = 0.98, P < 0.0001	Strain	712	F = 0.52, P = 0.4701
	Wild strain	W = 0.97, P < 0.0001			
	Males	W = 0.99, P = 0.1285	Sex	712	F = 0.64, P = 0.4248
	Females	W = 0.99, P = 0.0031			
	20˚C acclimation	W = 0.95, P < 0.0001	Acclimation	711	F = 28.17, P < 0.0001
	25˚C acclimation	W = 0.98, P = 0.0038			
	30˚C acclimation	W = 0.98, P = 0.0016			
	10 day olds	W = 0.97, P = 0.0001	Age	486	F = 14.71, P = 0.0001
	15 day olds	W = 0.98, P = 0.0041			
An. funestus CTmin (˚C)	ALL	W = 0.99, P = 0.0001	ALL	498	F = 3.51, P < 0.0001
	Laboratory strain	W = 0.95, P < 0.0001	Strain	520	F = 37.39, P < 0.0001
	Wild strain	W = 0.98, P = 0.0052			
	Males	W = 0.99, P = 0.0340	Sex	520	F = 0.03, P = 0.8723
	Females	W = 0.98, P = 0.0009			
	20˚C acclimation	W = 0.97, P = 0.0008	Acclimation	519	F = 39.68, P < 0.0001
	25˚C acclimation	W = 0.99, P = 0.2272			
	30˚C acclimation	W = 0.99, P = 0.1657			
	10 day olds	W = 0.95, P < 0.0001	Age	520	F = 0.28, P = 0.5947
	20 day olds	W = 0.97, P = 0.0002			
An. arabiensis CTmax (˚C)	ALL	W = 0.90, P < 0.0001	ALL	500	F = 7.24, P < 0.0001
	Laboratory strain	W = 0.92, P < 0.0001	Strain	522	F = 3.21, P = 0.0736
	Wild strain	W = 0.86, P < 0.0001			
	Males	W = 0.93, P < 0.0001	Sex	522	F = 72.28, P < 0.0001
	Females	W = 0.93, P < 0.0001			
	20˚C acclimation	W = 0.89, P < 0.0001	Acclimation	521	F = 2.65, P = 0.0716
	25˚C acclimation	W = 0.93, P < 0.0001			
	30˚C acclimation	W = 0.84, P < 0.0001			
	10 day olds	W = 0.87, P < 0.0001	Age	522	F = 4.72, P = 0.0302
	15 day olds	W = 0.92, P < 0.0001			
*An. funestu*s CTmax (˚C)	ALL	W = 0.88, P < 0.0001	ALL	516	F = 5.01, P < 0.0001
	Laboratory strain	W = 0.96, P < 0.0001	Strain	538	F = 1.42, P = 0.2339
	Wild strain	W = 0.81, P < 0.0001			
	Males	W = 0.97, P < 0.0001	Sex	538	F = 0.00, P = 0.9958
	Females	W = 0.77, P < 0.0001			
	20˚C acclimation	W = 0.69, P < 0.0001	Acclimation	537	F = 1.08, P = 0.3402
	25˚C acclimation	W = 0.97, P < 0.0001			
	30˚C acclimation	W = 0.96, P < 0.0001			
	10 day olds	W = 0.98, P = 0.0030	Age	538	F = 15.72, P < 0.0001
	20 day olds	W = 0.84, P < 0.0001			

An. arabiensis					
CTmin (˚C)	ALL	W=0.98, P < 0.0001	ALL	342	F = 5.75, P < 0.0001
Laboratory strain	Males	W=0.95, P < 0.0001	Sex	358	F = 0.33, P = 5673
	Females	W=0.98, P = 0.0029			
	20˚C acclimation	W=0.94, P < 0.0001	Acclimation	357	F = 9.65, P < 0.0001
	25˚C acclimation	W=0.98, P = 0.0705			
	30˚C acclimation	W=0.94, P < 0.0001			
	10 day olds	W=0.97, P = 0.0124	Age	357	F = 17.81, P < 0.0001
	15 day olds	W=0.92, P < 0.0001			
	20 day olds	W=0.87, P < 0.0001			
An. funestus					
CTmin (˚C)	ALL	W=0.94, P < 0.0001	ALL	392	F=3.67, P < 0.0001
Laboratory strain	Males	W=0.95, P < 0.0001	Sex	408	F=0.66, P = 0.4158
	Females	W=0.90, P < 0.0001			
	20˚C acclimation	W=0.91, P < 0.0001	Acclimation	407	F=64.83, P < 0.0001
	25˚C acclimation	W=0.98, P = 0.1214			
	30˚C acclimation	W=0.96, P = 0.0002			
	10 day olds	W=0.93, P < 0.0001	Age	407	F=31.57, P < 0.0001
	20 day olds	W=0.97, P = 0.0068			
	30 day olds	W=0.97, P = 0.0117			
An. arabiensis					
CTmin (˚C)	ALL	W=0.98, P < 0.0010	ALL	236	F=0.94, P = 0.5054
Wild strain	Males	W=0.99, P = 0.2095	Sex	246	F=8.04, P = 0.0050
	Females	W=0.97, P = 0.0033			
	20˚C acclimation	W=0.96, P = 0.0123	Acclimation	245	F=4.95, P = 0.0078
	25˚C acclimation	W=0.96, P = 0.0107			
	30˚C acclimation	W=0.97, P = 0.0274			
	10 day olds	W=0.96, P = 0.0018	Age	246	F=0.05, P = 0.8198
	15 day olds	W=0.97, P = 0.0136			
An. funestus					
CTmin (˚C)	ALL	W=0.98, P = 0.0052	ALL	220	F=1.82, P = 0.0524
Wild strain	Males	W=0.96, P = 0.0059	Sex	230	F=0.19, P = 0.6583
	Females	W=0.98, P = 0.1319			
	20˚C acclimation	W=0.98, P = 0.2028	Acclimation	229	F=1.48, P = 0.2296
	25˚C acclimation	W=0.98, P = 0.4414			
	30˚C acclimation	W=0.97, P = 0.1358			
	10 day olds	W=0.95, P = 0.0005	Age	230	F=9.94, P = 0.0018
	20 day olds	W=0.99, P=0.6761			
An. arabiensis					
CTmax (˚C)	ALL	W=0.93, P < 0.0001	ALL	423	F=4.12, P < 0.0001
Laboratory strain	Males	W=0.95, P < 0.0001	Sex	439	F=41.99, P < 0.0001
	Females	W=0.95, P < 0.0001			
	20˚C acclimation	W=0.92, P < 0.0001	Acclimation	438	F=0.84, P = 0.4309
	25˚C acclimation	W=0.93, P < 0.0001			
	30˚C acclimation	W=0.92, P < 0.0001			
	10 day olds	W=0.89, P < 0.0001	Age	438	F=2.75, P = 0.0653

	15 day olds	W=0.94, P < 0.0001			
	20 day olds	W=0.94, P < 0.0001			
An. funestus					
CTmax (˚C)	ALL	W=0.94, P < 0.0001	ALL	372	F=2.95, P < 0.0001
Laboratory strain	Males	W=0.89, P < 0.0001	Sex	388	F=6.73, P = 0.0098
	Females	W=0.98, P < 0.0072			
	20˚C acclimation	W=0.82, P < 0.0001	Acclimation	387	F=0.72, P = 0.4861
	25˚C acclimation	W=0.95, P = 0.0003			
	30˚C acclimation	W=0.97, P = 0.0009			
	10 day olds	W=0.98, P = 0.0269	Age	387	F=7.75, P = 0.0005
	20 day olds	W=0.96, P = 0.0010			
	30 day olds	W=0.88, P < 0.0001			
An. arabiensis					
CTmax (˚C)	ALL	W=0.85, P < 0.0001	ALL	232	F=7.36, P < 0.0001
Wild strain	Males	W=0.91, P < 0.0001	Sex	242	F=42.20, P < 0.0001
	Females	W=0.83, P < 0.0001			
	20˚C acclimation	W=0.82, P < 0.0001	Acclimation	241	F=13.58, P < 0.0001
	25˚C acclimation	W=0.92, P < 0.0010			
	30˚C acclimation	W=0.84, P < 0.0001			
	10 day olds	W=0.80, P < 0.0001	Age	242	F=0.38, P = 0.5382
	15 day olds	W=0.88, P < 0.0001			
An. funestus					
CTmax (˚C)	ALL	W=0.81, P < 0.0001	ALL	258	F=5.97, P < 0.0001
Wild strain	Males	W=0.97, P = 0.0070	Sex	268	F=0.76, P = 0.3835
	Females	W=0.69, P < 0.0001			
	20˚C acclimation	W=0.97, P = 0.0040	Acclimation	267	F=6.24, P = 0.0023
	25˚C acclimation	W=0.63, P < 0.0001			
	30˚C acclimation	W=0.97, P = 0.0861			
	10 day olds	W=0.97, P = 0.0166	Age	268	F=5.04, P = 0.0257
	20 day olds	W=0.73, P < 0.0001			
