# Supplementary material for: Thermal limits of wild and laboratory strains of two African malaria vector species, Anopheles arabiensis and Anopheles funestus
Source: Malar J. 2012 Jul 6;11:226. doi: 10.1186/1475-2875-11-226 (PMC3507762; doi:10.1186/1475-2875-11-226)
Supplement: Additional file 2 — Percentage deviation from the mean critical thermal minimum (CTmin) and maximum (CTmax) per group, per strain for Anopheles funestus and Anopheles arabiensis. [file 1475-2875-11-226-S2.pdf]

Additional file 2. Percentage deviation from the mean critical thermal minimum (CTmin) and maximum (CTmax) per group, per strain for *Anopheles funestus* and *Anopheles arabiensis*.

Strain and CT	Group	Mean CT (˚C)	% Deviation
An. funestus both strains	10 day olds	40.5	1.0
CTmax (˚C)	20 day olds	39.7	-1.0
	Males	39.7	-1.0
	Females	40.4	0.7
	20˚C acclimation	39.6	-1.2
	25˚C acclimation	40	-0.2
	30˚C acclimation	40.6	1.2
	wild	39.8	-0.7
	Lab	40.3	0.5
An. funestus both strains	10 day olds	8	-8.0
CTmin (˚C)	20 day olds	9.1	4.6
	30 day olds	9.1	4.6
	Males	9.1	4.6
	Females	8.4	-3.4
	20˚C acclimation	8.1	-6.9
	25˚C acclimation	8.5	-2.3
	30˚C acclimation	9.6	10.3
	wild	8.2	-5.7
	Lab	9.2	5.7
Laboratory *An. funestus*	10 day olds	40.7	0.7
CTmax (˚C)	20 day olds	40.1	-0.7
	30 day olds	40.2	-0.5
	Males	39.7	-1.7
	Females	41	1.5
	20˚C acclimation	39.8	-1.5
	25˚C acclimation	40.5	0.2
	30˚C acclimation	40.8	1.0
Wild *An. funestus*	10 day olds	40.2	1.0
CTmax (˚C)	20 day olds	39.4	-1.0
	Males	39.6	-0.5
	Females	40	0.5
	20˚C acclimation	39.3	-1.3
	25˚C acclimation	39.5	-0.8
	30˚C acclimation	40.6	2.0
Laboratory *An. funestus*	10 day olds	7.8	-15.2
CTmin (˚C)	20 day olds	10	8.7
	30 day olds	9.7	5.4
	Males	9.5	3.3
	Females	8.8	-4.3
	20˚C acclimation	8.5	-7.6
	25˚C acclimation	8.8	-4.3

	30˚C acclimation	10.2	10.9
Wild *An. funestus* CTmin (˚C)	10 day olds	8.2	0.0
	20 day olds	8.2	0.0
	30 day olds	8.2	0.0
	Males	8.5	3.7
	Females	7.9	-3.7
	20˚C acclimation	7.8	-4.9
	25˚C acclimation	8.3	1.2
	30˚C acclimation	8.8	7.3
An. arabiensis both strains CTmax (˚C)	10 day olds	40.1	1.0
	15 day olds	39.6	-0.3
	20 day olds	39.5	-0.5
	Males	38.9	-2.0
	Females	40.6	2.3
	20˚C acclimation	39.8	0.3
	25˚C acclimation	39.3	-1.0
	30˚C acclimation	40.1	1.0
	wild	39.8	0.3
	Lab	39.7	0.0
An. arabiensis both strains CTmin (˚C)	10 day olds	9.8	0.0
	15 day olds	9.6	-2.0
	20 day olds	10.1	3.1
	Males	10.2	4.1
	Females	9.5	-3.1
	20˚C acclimation	9.1	-7.1
	25˚C acclimation	10	2.0
	30˚C acclimation	10.3	5.1
	wild	8.8	-10.2
	Lab	10.9	11.2
Laboratory *An. arabiensis* CTmax (˚C)	10 day olds	40.1	1.0
	15 day olds	39.5	-0.5
	20 day olds	39.5	-0.5
	Males	38.7	-2.5
	Females	40.6	-2.3
	20˚C acclimation	39.7	0.0
	25˚C acclimation	39.6	-0.3
	30˚C acclimation	39.7	0.0
Laboratory *An. arabiensis* CTmin (˚C)	10 day olds	11.2	2.8
	15 day olds	10.7	-1.8
	20 day olds	10.8	-0.9
	Males	11.2	2.8
	Females	10.5	-3.7
	20˚C acclimation	10	-8.3
	25˚C acclimation	11.5	5.5
	30˚C acclimation	11.1	1.8

Wild *An. arabiensis* CTmax (˚C)	10 day olds	40.2	1.0
	15 day olds	39.7	-0.3
	20 day olds	39.5	-0.8
	Males	39.1	-1.8
	Females	40.5	1.8
	20˚C acclimation	40	0.5
	25˚C acclimation	39	-2.0
	30˚C acclimation	40.5	1.8
Wild *An. arabiensis* CTmin (˚C)	10 day olds	8.5	-3.4
	15 day olds	8.5	-3.4
	20 day olds	9.4	6.8
	Males	9.1	3.4
	Females	8.5	-3.4
	20˚C acclimation	8.3	-5.7
	25˚C acclimation	8.6	-2.3
	30˚C acclimation	9.5	8.0
